# Understanding and applying biological resilience, from genes to ecosystems

**DOI:** 10.1038/s44185-023-00022-6

**Published:** 2023-08-28

**Authors:** Rose Thorogood, Ville Mustonen, Alexandre Aleixo, Pedro J. Aphalo, Fred O. Asiegbu, Mar Cabeza, Johannes Cairns, Ulrika Candolin, Pedro Cardoso, Jussi T. Eronen, Maria Hällfors, Iiris Hovatta, Aino Juslén, Andriy Kovalchuk, Jonna Kulmuni, Liisa Kuula, Raisa Mäkipää, Otso Ovaskainen, Anu-Katriina Pesonen, Craig R. Primmer, Marjo Saastamoinen, Alan H. Schulman, Leif Schulman, Giovanni Strona, Jarno Vanhatalo

**Affiliations:** 1 HiLIFE Helsinki Institute of Life Science, University of Helsinki, Helsinki, Finland; 2 Research Programme in Organismal & Evolutionary Biology, Faculty of Biological and Environmental Sciences, University of Helsinki, Helsinki, Finland; 3 Department of Computer Science, Faculty of Science, University of Helsinki, Helsinki, Finland; 4 Helsinki Institute for Information Technology, University of Helsinki, Helsinki, Finland; 5 Institute of Biotechnology, HiLIFE Helsinki Institute for Life Science, University of Helsinki, Helsinki, Finland; 6 LUOMUS Finnish Museum of Natural History, University of Helsinki, Helsinki, Finland; 7 Viikki Plant Science Centre, University of Helsinki, Helsinki, Finland; 8 Department of Forest Sciences, Faculty of Agriculture and Forestry, University of Helsinki, Helsinki, Finland; 9 HELSUS Helsinki Institute of Sustainability Science, University of Helsinki, Helsinki, Finland; 10 CE3C - Centre for Ecology, Evolution and Environmental Changes, CHANGE—Global Change and Sustainability Institute, Faculty of Sciences, University of Lisbon, 1749-016 Lisbon, Portugal; 11 Research Programme in Ecosystems and Environment, Faculty of Biological and Environmental Sciences, University of Helsinki, Helsinki, Finland; 12 BIOS Research Unit, Helsinki, Finland; 13 Research Centre for Ecological Change, Faculty of Biological and Environmental Sciences, University of Helsinki, Helsinki, Finland; 14 Syke Finnish Environment Institute, Helsinki, Finland; 15 SleepWell Research Program, Faculty of Medicine, University of Helsinki, Helsinki, Finland; 16 Department of Psychology and Logopedics, Faculty of Medicine, University of Helsinki, Helsinki, Finland; 17 Neuroscience Center, HiLIFE Helsinki Institute for Life Science, University of Helsinki, Helsinki, Finland; 18 VTT Technical Research Centre of Finland Ltd, Espoo, Finland; 19 Department of Evolutionary and Population Biology, Institute for Biodiversity and Ecosystem Dynamics, University of Amsterdam, Amsterdam, The Netherlands; 20 Natural Resources Institute Finland (Luke), Helsinki, Finland; 21 Centre for Biodiversity Dynamics, Department of Biology, Norwegian University of Science and Technology, Trondheim, Norway; 22 Department of Biological and Environmental Science, University of Jyväskylä, Jyväskylä, Finland; 23 European Commission, Joint Research Centre, Directorate D – Sustainable Resources, Ispra, Italy; 24 Department of Mathematics and Statistics, Faculty of Science, University of Helsinki, Helsinki, Finland; 25 Present Address: Onego Bio Ltd, Helsinki, Finland

**Keywords:** Ecology, Biodiversity, Community ecology, Ecological modelling, Ecosystem ecology, Evolutionary ecology, Molecular ecology, Palaeoecology

## Abstract

The natural world is under unprecedented and accelerating pressure. Much work on understanding resilience to local and global environmental change has, so far, focussed on ecosystems. However, understanding a system’s behaviour requires knowledge of its component parts and their interactions. Here we call for increased efforts to understand ‘biological resilience’, or the processes that enable components across biological levels, from genes to communities, to resist or recover from perturbations. Although ecologists and evolutionary biologists have the tool-boxes to examine form and function, efforts to integrate this knowledge across biological levels and take advantage of big data (e.g. ecological and genomic) are only just beginning. We argue that combining eco-evolutionary knowledge with ecosystem-level concepts of resilience will provide the mechanistic basis necessary to improve management of human, natural and agricultural ecosystems, and outline some of the challenges in achieving an understanding of biological resilience.

## Introduction

The Anthropocene is characterised by the pervasive impact of human activity on all aspects of life on earth^1^. Human-driven climate change and overexploitation of natural resources, as well as increasing human population densities and urbanisation, are placing progressively larger areas under human influence and disturbances such as increased and/or more variable temperatures (and associated events such as droughts and fires), direct anthropogenic alterations (e.g. pollution, land-use changes, habitat fragmentation), and introduction of invasive species^2^. Even the world’s topology has changed, as global movement of individuals and goods erodes biogeographical barriers^3^. These environmental changes put ecosystems under unprecedented and accelerating pressures, inducing regime shifts^4^, causing loss of ecosystem services^5^, and even changing the course of evolution^6^. There is therefore an urgent need to determine why some species, communities or ecosystems decay while others persist or adapt^7^, and then implement this knowledge for improved management practices that can reverse or mitigate damage^8^.

**Fig. 1:**
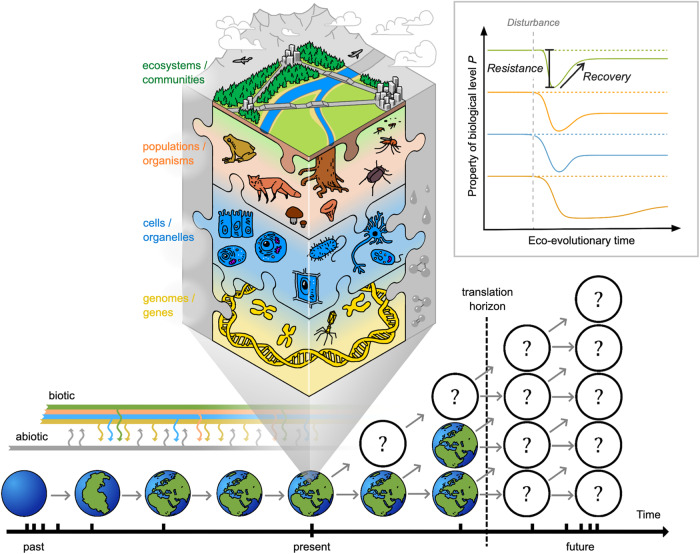
Biological resilience (mechanisms and processes across biological levels that enable systems to resist disturbance and/or recover over time back to a steady state after perturbations) is mediated by connections within and among levels of organisation (simplified to genes and genomes, cells and organelles, organisms and populations, communities and ecosystems; depicted by multi-coloured shading and lines), and recognises that the present state (expanded in centre of figure) is shaped by ecological and evolutionary responses to past biotic (multi-coloured) and abiotic (grey) disturbance and selection (note that time is represented by a log-scale). Resistance (change) and recovery (time, state and rate) can be measured using properties of different biological levels (inset) to provide a ‘common currency’ for integration, and then enhance the translation horizon (vertical dashed line, close in time) by providing more readily measurable indicators and improving accuracy of forecast outcomes (grey arrows and question marks within circles). Note that the resistance and recovery trajectories of biological levels to a disturbance event may differ in both amplitude and temporal scale (inset), and that ‘recovery’ is also sometimes referred to in the literature as a measure of resilience (e.g. refs. ^11,27^).

In ecology, ‘resilience’ has attracted great interest as a concept that describes the capacity of a system to respond to disturbance (Table 1, Fig. 1 inset, following ref. ^9^; see ref.s^10,11^ for recent in-depth reviews of definitions). Ecosystems may show strong ‘resistance’ with minimal perturbation in state or function. Or, if perturbed (i.e. low resistance), ecosystems may over time ‘recover’ and move back towards their previous state, or even benefit from the disturbance. Systems with low recovery potential, on the other hand, may shift abruptly (i.e. a tipping point) into a new and possibly stable state (i.e. a regime shift). Resilience has therefore typically been studied theoretically and empirically by considering how a system returns to its previous state (‘engineering resilience’^12^) or by the amount of disturbance absorbed before it tips into a different state (‘ecological resilience’^13^). However, translating the concept of resilience into an understanding of the mechanisms or properties that determine how much an ecosystem can absorb or resist a disturbance, or what shapes the trajectory of its recovery back to a previous or new stable state, remains challenging^10,14^. In part, this may be because resilience has typically been studied at the level of the ecosystem^15,16^ which reduces our power to identify how and why resistance and/or recovery responses occur^17^: understanding the behaviour and interactions of a system’s component parts is essential to understand and forecast ecology^18^. On the other hand, while studying lower biological levels in isolation makes it easier to measure properties that might comprise a system’s resilience (e.g. population size, individual fecundity, genetic diversity; see Fig. 1 inset), reductionist approaches can hinder detection of connections between seemingly isolated biological events^19^. How can we deal with this complexity to identify the critical drivers and indicators of resistance and recovery?

**Table 1:** Definitions and examples of key terminology and how used across biological levels

Term	Definition - resilience in ecology	Definition or use at biological levels	Examples relevant for biological resilience
**Disturbance event**	abiotic or biotic force, process, or agent with potential to impact a system	equivalent but not often used as a defined term. Somewhat analogous to a selection event	• broad scale e.g. Climate change & local scale e.g. introduction of invasive species • short to long duration, or pulses • multiple disturbances are possible, related directly (e.g. temperature & drought) or indirectly (e.g. eutrophication & invasive species) • can be experimentally approximated in the field and/or lab
**Perturbation**	response of a system to a disturbance, measured as change in a state variable	alteration of function	• sometimes synonymous with ‘disturbance’ • ‘Perturbation biology’ concerns changes in proteins and cellular features, modelled using networks^121^ • e.g. gene knock-out studies as perturbation to multiple factors^122^, conservation translocations of social phenotypes^87^
**Resilience**	capacity of a system to manage disturbance	somewhat analogous to homoeostasis, a self-regulating feedback process that maintains physiological stability	• where used, resilience can be a metaphor, property of dynamic models, or a measurable quantity • broad adoption of resilience including socioecological systems, neurobiology, psychology, medicine • disruption of homoeostasis leads to disease, i.e. a state-change^123^
**Resistance**	ability to persist despite a disturbance event	preventing infection or invasion by an enemy; includes physical, behavioural or cellular defences	• cellular ‘memory’ from past exposure influences drug resistance of cancer cells^124^ • behavioural defences against parasites vary according to social structure^125^
**Recovery**	ability to return over time towards a pre-disturbance state	somewhat analogous to tolerance, ability to maintain fitness despite e.g. infection, lack of resource	• measured as time to recover, amount of recovery, and rate of recovery^11^ • physiological drought-tolerance as mechanism for resilience of grasslands to climate change^126^
**Plasticity**	not commonly used, but a potentially important mechanism shaping resistance and recovery components of resilience?	variation in the expression of a gene/trait due to differences in environmental conditions	• gene expression plasticity and stress tolerance^127^ • physiological plasticity and resilience to climate warming^46^ • behavioural plasticity and resilience of communities^128^

Here we propose that this can be achieved by adopting a ‘biological resilience’ framework (Fig. 1) where we: (1) test ecosystem-level resilience concepts (i.e. resistance and recovery responses, state changes) across lower levels of biological organisation; and (2) harness knowledge provided by the eco-evolutionary history of adaptation to past perturbations to better understand resilience from the bottom up. In doing so, biological resilience acknowledges that processes occurring within and between components across biological levels, from genes to communities, shape how systems resist disturbance or recover from perturbations. This framework stands out from recent calls to encourage analysis of resilience across systems and scales, and from ecosystems to populations (e.g. refs. ^16,17^) as we explicitly acknowledge the crucial role of eco-evolutionary history. Furthermore, investigating how biological levels themselves respond over time to a disturbance event (e.g. from changes in cellular processes to genetic adaptation via measures of gene or allelic diversity) would provide scope for a common ‘language’ and integration of data to dive deeper into uncovering the mechanisms and processes that afford resilience from individuals to communities and ecosystems. We first (i) explore how the eco-evolutionary past provides context for present and future resistance and recovery responses, and then (ii) discuss why it is necessary to consider how abiotic and biotic disturbance events can affect biological levels differently to detect mechanisms and underlying processes. Next, we (iii) outline three testable hypotheses to kick-start research into resilience across levels of biological organisation, from genes to cells, individuals, populations and communities. Collecting and integrating large amounts of data about how every biological component responds to a disturbance is often considered unrealistic. However, here we (iv) identify new opportunities emerging from the ongoing infusion of big data into ecology and evolutionary biology and stress the need to combine these data with experimental approaches to (v) enable advances in translating research into practice. Each of these steps is beginning to be investigated (examples across taxa, biological levels, and ecological context are given throughout) but they lack an overarching framework that brings all of them together. Our aim here is therefore not to cover all aspects of how resilience is, or could be, studied in-depth, but to extend recent calls to move from ecosystems to species (e.g. ref. ^16^) and encourage discussion of why and how ‘resilience thinking’ could be applied across biological levels.

## (Re)Placing resilience into an eco-evolutionary context

When Holling introduced ecological resilience in his landmark paper^13^, he briefly suggested that a system’s resilience is a product of its evolutionary history (1973:p.18). Most research conducted since, however, has lacked an evolutionary perspective^14,20^. Therefore, much of the discussion, theory and examples of resilience in ecology lack a long time horizon and largely ignore how past environments influence current (or future) resistance and recovery responses^21^. Similarly, eco-evolutionary biologists rarely study how a system’s resilience might be conferred by processes that occur within or across the biological levels that form the focus of their studies^22^, despite research programmes often having a shared interest in determining how particular measurable traits or variables vary in response to a stressor or disturbance event (e.g. ref. ^23^). This disconnect among fields may be because much of the work on resilience describes patterns at the ecosystem level^14^, whereas studies of evolutionary processes rarely scale to complex communities^24^. Indeed, focusing on how ecology and evolution shape patterns and processes within individuals and populations has attracted criticism for being too narrow to address large ecological problems^20,25^. Nevertheless, here we argue that adopting a ‘common currency’ of studying resistance and recovery across biological levels will improve integration of eco-evolutionary theory with resilience (see Box 1) and provide information from the evolutionary past to improve our power to estimate both present and future states.

Box 1 Integrating ecology and evolution to understand biological resilience Evolutionary mechanisms (mutation, drift, migration, natural selection) generate changes in allele frequencies from one generation to another (i.e. microevolution) and, given sufficient time or conditions, can lead to large-scale changes that transcend species boundaries (i.e. macroevolution). Similarly, processes that influence ecology (e.g. density, connectivity, competition, species interactions) at smaller scales (e.g. within populations, communities) give rise to large-scale macroecological patterns (e.g. biodiversity and ecosystem function). Darwin made no distinction between micro and macro scales, nor did he (or Wallace) separate ecology from evolutionary processes (see ref. ^20^). Over the 20th century, however, research in ecology and evolution specialised to specific scales and processes that presents a major challenge for understanding ecological patterns and processes^20^. Adopting a biological resilience framework necessitates reintegration. How might this be achieved?

**(i) Harness existing and emerging approaches**

The combination of theory, modelling and empirical approaches of eco-evolutionary dynamics provides a potential solution to reintegrate ecological and evolutionary processes across biological levels and scales^85,136,137^ and detect relevant responses to environmental change (e.g. refs. ^23,138^). Here, phenotypic and genotypic variation coupled with rapid evolution play a key role to explain how populations scale up to influence species interactions and ecological communities (including their structure, function, and dynamics), as well as influence how selection pressures are responded to and genomes are inherited. Work in this rapidly developing field is scaling up from population-level studies^139^ to analyse how evolutionary processes impact ecological dynamics (and vice versa) in communities and even ecosystems^136^, with explicit acknowledgement that interactions and feedback also occur across non-adjacent biological levels (see Fig. 1 in refs. ^136,140^, and see ref. ^141^ for a review of available models) – as we propose here for biological resilience. Studies of eco-evolutionary dynamics are possible in both the lab and the field^85^ and are expanding in scope towards a landscape perspective^24,51,142^. Taking an eco-evolutionary approach to consider feedbacks on ecosystem-level processes is also now beginning to attract attention, suggesting that evolutionary changes in the variation of traits may play an important role in shaping how and when ecosystems reach tipping points and possibly irreversible ecosystem change^22^.

**(ii) Recognise conceptual similarities**

Understanding biological resilience will require a step change to move from describing either macro- or micro- scale patterns to demonstrating how evolutionary and ecological processes shape short- and longer-term responses to environmental change. Fortunately, eco-evolutionary dynamics and resilience in ecology rely on similar landscape-based frameworks to conceptualise and mathematically explore predictions. Resilience is typically described by a ‘stability landscape’ where valleys in the landscape represent alternative stable states and disturbance events create wobbles that may push systems over the hilltops between valleys (see ref. ^10^). In principle, these landscapes can be described by mathematical functions and may be measured by identifying the critical state variables that describe its dimensions, although in practice it remains very challenging to identify alternative stable states available in the past or the future^10^. Similarly, evolutionary biology makes use of ‘adaptive landscapes’ where fitness functions are described according to phenotypic traits (or genotypes) to conceptualise and predict the strength and direction of selection. Populations or species are described as ‘climbing’ towards ‘adaptive peaks’ of trait/genotype combinations with the highest fitness, where ‘adaptive valleys’ of lower fitness inhibit movement across the landscape. Emerging topics of research include integrating environmental variables to understand past, current and future movement among adaptive peaks (e.g. refs. ^143,144^). Although the axes of adaptive and resilience landscapes are at different biological levels (typically populations and ecosystems, respectively) and the location of stable states are inverse (‘peaks’ in adaptive landscapes, ‘valleys’ in resilience landscapes), in both cases the population or system of interest is expected to oscillate and move across the landscape in response to ecological change. Considerable effort is now going into translating these landscapes from metaphor to useful predictive tools (e.g. resilience landscapes^10^, adaptive landscapes^143^) meaning the time is right to bridge the gap.

**(iii) Identify shared terminology**

A lack of common language is a widely recognised barrier to disciplinary integration, and this is further exacerbated when fields share jargon but differ in definitions, or when definitions of key terms are easily confused (e.g. ref. ^145^). This is a problem for integrating evolutionary biology with resilience across biological levels as ecologists and evolutionary biologists share terms but use them differently (Table 1). For example, ‘resistance’ is used in resilience (e.g. ref. ^11^) to describe how much a system is perturbed by a disturbance event (i.e. a rate) whereas biologists studying pathogens, parasites and antagonistic coevolution define resistance as a strategy to prevent or limit infection by an enemy (i.e. a trait, or suite of traits; e.g. ref. ^146^). ‘Tolerance’ on the other hand describes how much a host can prolong its survival or recover its reproductive success, given infection^147^. This perhaps has analogies to ‘recovery’ back to a stable state in resilience (see Introduction, Fig. 1), although time to recover is less of a focus in studies of tolerance than resilience. Providing in depth equations is beyond the scope of this conceptual overview, and even amongst existing studies of resilience, there is variation in how resistance and recovery parameters are measured (see ref. ^11^ for an overview of studies of resilience in forest trees, soil communities, and watersheds). The first step for comprehensive studies utilising information across disciplines is therefore to build a shared glossary, preferably in mathematical terms that relate to the landscapes outlined in (ii), with expectations of how (and when) putative state variables at the different biological levels being measured will respond to the disturbance event of interest.

## Using eco-evolutionary theory to read the past from the present state

Estimating components (or attributes) of resilience such as resistance and recovery rely on measures before, during and after disturbance events (see ref. ^11^ for example equations used in different ecological contexts). This presents a major challenge for understanding resilience, as even if the ‘before disturbance event’ state is contemporaneous or known, it is rare that information is available about what stable states may have been like in the past. Evolutionary genetics provides an approach to help tackle this problem, as past perturbations leave their mark on the genome (i.e. ‘evolutionary memory’^26^) which can (i) affect an individual’s capacity to respond, (ii) influence a population’s ability to adapt to changing environmental conditions, and therefore (iii) shape ecological community interactions and potentially ecosystem function now and in the future, even if perturbations are novel to those experienced in the past^27^. At the level of genes, evolutionary history is manifested in variation introduced by mutation and/or migration (gene flow) as well as recombination (new combinations of genetic variation) that is filtered by natural selection or fixed by random genetic drift. Some genetic variants may provide an advantage against future disturbance events, such as through acquired resistance against a parasite, pest or antibiotic encountered in the past^28^. On the other hand, disturbances that result in severe population bottlenecks can result in the loss of potentially beneficial variation and/or fixation of maladapted alleles, and thus have negative effects on resilience^29^. Similarly, past selection that strongly favoured specific alleles may also limit future resilience due to the loss of genetic variation required for new adaptation to take place (e.g. Afrotropical butterfly experiencing climate change induced variation in seasonality^30^).

The principle of evolutionary parsimony states that species with a shared evolutionary history are likely to have experienced similar selection pressures (e.g. from shared disturbance events) in the past^31^, and therefore possibly convergent responses at different biological levels. It is perhaps not surprising, then, that a recent study harnessing evolutionary history found that current variation in demographic resilience (i.e. responses of population growth and size) was explained more by phylogenetic relatedness among species than recent (~50 years) environmental stochasticity^21^. Incorporating historical global temperature records, species-level functional traits, and rates of phylogenetic diversification is also helping to explain how microevolutionary history induces different macroevolutionary responses to temperature change across angiosperms^32^. Evolutionary history can also be harnessed to understand resilience at the cellular and molecular level, with comparisons of e.g. protein interactomes across the tree of life revealing how these complex networks of molecular interactions evolve greater resilience to a loss of network connections over time^33^. Moving beyond phylogenetic relatedness, there is a rich body of evolutionary theory (e.g. the Coalescent) and simulation frameworks (e.g. SLiM) available to estimate past population sizes and genetic diversity (summarised in ref. ^34^) or the prevalence of deleterious genetic mutations in response to dated environmental events (e.g. ref. ^35^). These tools could be used to model resistance and recovery of populations or species of interest to current and future disturbance scenarios (e.g. ref. ^36^ and see ref. ^37^ for a workflow to detect and predict responses to thermal disturbances), or by comparing demographic histories for interacting species (see ref. ^34^ for an example with great apes, malaria *Plasmodium*, and the *Anopheles* mosquito vector), it could soon be possible to gain a deeper perspective on past states of biological levels from populations and single species to communities and ecosystems.

While genetic information will underpin the capability of an organism to respond, there is now also abundant evidence from many taxa that genotypes can generate different phenotypic (including cellular, physiological, morphological, and behavioural) responses depending on environmental conditions (i.e. plasticity, Table 1). Such plasticity can enable individuals to resist negative impacts on fitness and consequently buffer (or even increase) populations from possible demographic perturbations (see ref. ^38^), or be maladaptive if it leads individuals to respond inappropriately to previously reliable environmental cues (e.g. ref. ^39^). Phenotypically plastic responses can be modified further depending on the composition, structure and spatial context of the perturbed population or ecological community^40^, and variation in plasticity can influence individual and species interactions and therefore feedback on community composition and ecosystem function^41,42^. Although often studied by measuring individual phenotypes or gene expression in response to a specific environmental condition (i.e. representing a disturbance event), it is now acknowledged that plasticity may leave heritable ‘epigenetic’ marks on the genome (i.e. not changes to DNA sequences) that influence the future regulation of gene expression and shape how subsequent generations may resist or recover (e.g. refs. ^43,44^). Therefore, phenotypic plasticity that evolved in the past may be ‘read’ now to explain current, and predict future, states and interactions across biological levels. While there is growing interest in testing whether current plasticity plays a significant role in resistance and recovery to e.g. climate warming (heterotrophy of corals^45^, physiology of ectotherms^46^, demographic variation of commercially-important fishes^47^), there have been few attempts to ‘read the past’ from current plasticity^38,48^. Harnessing knowledge about the past to understand biological resilience will likely require integrating phenotypic plasticity, epigenetics and genetic information (e.g. ref. ^49^), meaning there is potential to provide a major advance across diverse fields.

## Finding the right scale: effects of disturbance events vary across biological levels

If we can uncover how elements of the system have responded to past disturbance events or state perturbations, then this information will become useful for predicting current and future changes. However, disturbances can be complex and vary in intensity, duration, frequency and spatial extent^50^ and the impact of disturbance events on both the degree and timing of any perturbation will vary across biological levels (Fig. 1). For example, an adaptive genetic mutation^51^ or socially-inherited behaviour^52^ enabling a species to exploit its perturbed habitat can in turn, alter community assembly through variation in demography. This may or may not occur contemporaneously with the spread of the genetic mutation, as community changes caused by past disturbances may also determine subsequent community assembly through complex cascading effects on species succession (e.g. the order in which species recolonize an area after a habitat perturbation is important for community assembly^53^) and potentially ecosystem function. Adopting a biological resilience framework could help to predict these events as incorporating a longer time horizon reveals resilience to be a dynamic and constantly evolving product of long term (co-) evolutionary, ecological and biogeographical processes (e.g. ref. ^54^).

Understanding how these processes operate at different biological levels of organisation will be critical, as the rate of evolution for example is constrained by generation times that vary from minutes (e.g. cells and microbes) to centuries (e.g. trees), reproductive strategy influences opportunities for outcrossing and mutation, and migration can diversify or limit local genotypic and phenotypic variation. However, at present, it remains unclear whether one level in particular will be of greater importance for predicting responses to current and future disturbance, and while it is likely that responses of one level to a given disturbance event will influence how multiple other levels respond, investigations into the carry-over effects of perturbations across biological levels are few and mostly focus on adjacent levels (e.g. changes in population influence response of communities^55^). The composition, structure and spatial context of a perturbed population or ecological community also needs to be taken into account^40^. Range-edge populations, for example, can be comprised of a different set of individual response-types than those found in the range core (e.g. spatial sorting^56^) and potentially set up cascades of change across other biological levels (e.g. reduced genetic diversity^57^), and fragmented habitats influence the degree to which species can reduce their exposure to perturbations by shifting, shrinking or expanding their range via dispersal^58^, or by modifying physiological or behavioural responses^59^. Spatial context also has fundamental implications for longer-term adaptation to environmental change as it shapes gene flow^60^. Integrating past and present distributions and habitats is therefore likely to be a key, albeit challenging, aspect to understand biological resilience. Nevertheless, using evolutionary history as a ‘natural experiment’ and integrating information about adaptation explicitly into a resilience framework could provide a previously untapped resource for predicting how ecological systems respond to disturbance events.

## A biological resilience framework generates testable hypotheses

It is clear that determining how different biological levels resist and recover and buffer other levels from perturbations will be complex, and that harnessing available information from the past is not straightforward. However, theory and mathematical models lay the foundations for identifying what to measure from experimental and empirical systems and how to extract these observations from real data (Box 1). Much of the theoretical work on resilience has made use of complex dynamic system models (e.g. ref. ^61^), but simpler approaches to calculate resilience are available (e.g. ref. ^15^), and efforts to incorporate evolutionary perspectives into models of ecosystem-level responses (e.g. tipping points^22^, warning signals^54^, species coexistence^62^) and model complex interactive processes across biological levels (e.g. network models^63^) are beginning. Furthermore, there is growing theory surrounding the ecological and evolutionary dynamics of resistance (e.g. antibiotics^64^) and rapid genetic adaptation to ecological change (e.g. ref. ^65^) that could provide useful approaches to bridge resistance and recovery responses across biological levels. A long-term problem in ecological modelling, however, is that theoretical models are good for understanding causality, but difficult to test critically with data, whereas statistical models are correlative, and thus may not identify the relevant underlying mechanisms even if they fit the present data well. Nevertheless, considering perturbations across biological levels in terms of eco-evolutionary form and function helps generate hypotheses concerning the role of past disturbances in shaping current and future resilience (i.e. resistance and recovery, Fig. 1): (i) past experience primes a biological entity to cope with future disturbances of a similar nature. Alternatively, but not necessarily mutually exclusively, (ii) populations and communities exposed to more variable environments and higher levels of disturbance over the long term are expected to be most resilient. However, even these may accrue a resilience debt if the magnitude and frequency of the disturbances differ too much from their historical disturbance regimes^66^. Finally, (iii) even without long-term disturbance histories, rapid adaptation may improve resilience against specific stressors. This may, however, come at the cost of decreased resilience in the longer term because of reduced pre-existing diversity after rapid adaptation or altered species interactions^57,67^. Aspects of these hypotheses have already begun to be tested (Table 2), but not yet across biological levels within a relevant system.

**Table 2:** Three hypotheses regarding how the ecological and evolutionary past shapes current and future responses to environmental change, and the multiple study approaches required to understand this biological resilience (with examples)

Hypotheses	Methodological approaches	Examples
**(i) past experience primes a biological entity to cope best with future disturbances of a similar nature**		
	Describe patterns using correlational or before-after survey data	• Current and future responses are mediated by past infection using long-term data on Soay sheep^129^ • Co-occurrence of taxa before and after Holocene^83^
	Use modelling and simulations to generate testable predictions	• Transgenerational priming^77^
	Perform experimental perturbations in micro- or mesocosms or field settings	• Experimental evolution with yeast^76^ • Legacy effects of drought exposure on microbial communities^130^ • Transgenerational acquired resistance in model plants^43^ • Resurrection studies^93^
	Interrogate findings with data from natural experiments	• Captive and wild songbirds respond differently to temperature perturbations^78^
**(ii) diversity of environments and disturbances in the past generates greater resilience in the future**		
	Make use of long-term survey data and/or big ecological and genetic datasets (including ancient DNA) to measure past diversity	• Paleological history^131^ • Ecological and evolutionary memory^33,66^ • Adaptive genetic diversity^57^
	Use modelling and simulations to generate testable predictions	• Predicting a species response to environmental change when preadaptation of community differs^132^
	Perform experimental perturbations in micro- or mesocosms or field settings	• Resurrection studies^93^
	Interrogate findings with real-world examples, e.g. natural experiments	• Biological invasions^133^
**(iii) rapid adaptation to match current conditions reduces future resilience**		
	Compare current resilience of biological entities and search for signs of rapid adaptation in the past	• Genome-wide scans in forest trees to detect adaptation to aridity^134^
	Use modelling and simulations to generate testable predictions	• Evolutionary rescue^135^
	Experimentally induce a novel perturbation in cases where rapid adaptation is present vs. absent	• Resurrection studies^93^

## Approaches to understand biological resilience

Understanding biological resilience will require concerted multidisciplinary research programmes where the effects of a disturbance (or multiple stressors) in terms of resistance and recovery responses are investigated across different levels, and where feedback among levels is also measured explicitly (Table 2, Fig. 1). At present, research into coral reef resilience provides a worked example: surveys and experiments have demonstrated that different coral species exhibit different degrees of resistance and recovery to similar stressors^68^. Comparing the species’ evolutionary history provides some insight into why: a recent study suggests Caribbean corals show lower recovery than Indo-Pacific corals due to an evolutionary bottleneck 2.8 million years ago that favoured large and long-lived species with low rates of recruitment^69^. Efforts to investigate genomic predictors of coral bleaching^70^, and even to assist evolution towards more resilient forms^71^, are also now attracting wide attention^72^. Furthermore, mapping dependencies of coral-fish species based on natural history and fitting structural equation models has recently suggested that coral loss may lead to substantial negative change in fish diversity and biomass worldwide, with effects extending beyond the fish species directly dependent on corals^55^. Salmonid fishes (see Box 2) could also provide a model system for similar combinations of approaches to better understand current changes in populations following disturbances (including at the ecosystem level) from fishing, find reliable indicators of the mechanisms that improve recovery, and provide more reliable forecasts of management scenarios.

There are many other studies beyond these examples that report genetic-, phenotypic-, or community-level changes along environmental gradients or responses to natural changes, but far fewer either consider more complex environmental scenarios (e.g. multiple or sequential stressors) or how the effects at one biological level may affect others. As such, much of the current work in understanding biological resilience (even if not yet couched in this terminology) relies on surveys and correlations that are carried out at one level. For example, ‘which genes contribute to more resilient phenotypes?’^73^, ‘which populations are more resilient to certain perturbations?’^74^ or, ‘which species are most affected by which particular aspects of a perturbation?’^75^. Furthermore, the results of experiments, particularly into resilience at the cellular^76^ or genetic levels^77^, are often not interpreted in a broader ecological context or compared to available data from natural populations^78^. Here we explore how we can move beyond studying the effects of single stressors or single species or levels and progress towards more complex experimental designs and assessments of more complex situations in the wild. Although this survey is not exhaustive, we hope that it provides insight into the range of methodologies used across biological levels to better enable discussion and design of multidisciplinary research.

To enable future studies to cover multiple biological levels, incorporating standardized collection of data and sample material across biological levels (e.g. genetic material, phenotype and community structure) into geographical surveys and long-term studies is a good starting point. If these standardised surveys are conducted over multiple seasons, years, or generations, this long-term monitoring has the potential to facilitate (i) detection of subtle responses and/or subtle perturbations, (ii) replication over time, and (iii) detection of ecological and evolutionary memories^79^. The same recommendation is relevant for “opportunistic” sampling following the (often unexpected) formation of a resilience-relevant gradient/difference. Data for multiple biological levels at sites that have experienced a heat wave for example, or an oil spill or chemical release, can either be compared to those of a nearby site that did not experience the perturbation^80^, or in the event that surveys of the affected sites were conducted prior to the perturbation, a ‘before vs. after’ analysis can be conducted^81^. Second, the prehistoric and palaeocological record is an important potential source of survey data, as it is now becoming tractable to incorporate with extant data (e.g. biotic interactions through food web analyses, process-based models of origin and extinction, and species co-occurrence matrices, ref. ^82^). This paleo-perspective could offer natural experiments: data are available to potentially help explain how community assembly (and disassembly) works when time spans are increased^83^, for example, or how genetic structure and adaptations respond to perturbations ranging from major extinctions to rapid climate change or species invasions over long time periods (e.g. ref. ^84^).

A major challenge for survey approaches mentioned above however is to disentangle the effects of co-varying environmental characteristics (e.g. photoperiod and temperature along a latitudinal gradient, or simultaneous drought and reduced food availability). Therefore, experiments in semi-natural (e.g. in vitro microcosms or outdoor mesocosm setups) or field settings (e.g. ponds/tanks, forest/field plots, enclosures suitable for small mammals, or free-ranging individuals and populations) are an essential third approach to test how resilience occurs across biological levels, and offer an attractive compromise where ‘real-world’ conditions are partly retained but where some manipulation and/or control is nevertheless possible, together with replicates^85^. These experiments can range greatly across organismal scale, geography, and biological levels (e.g. ref. ^48,86^), and can also be conducted alongside interventions to mitigate species decline or change in ecosystem function (e.g. conservation actions including introductions of individuals or translocations of populations^87^), if the selection of individuals or species to be moved is designed to test the relative resilience of different characteristics (e.g. social behaviour^88^, genetic diversity^67^). Although further removed from ‘real world’ conditions, common garden experiments (i.e. the rearing individuals in a controlled environment under common conditions) could be used to study responses to environmental or anthropogenic stressors by adding ‘treatments’ such as thermal stress, disease, or changes in community (e.g. flour beetles^89^, burying beetles^90^). Here, environmental differences can be eliminated, or specific environmental factors can be tested so that the extent of resilience that is plastic versus evolutionary (e.g. fish^91^, crops^92^) can be measured. Resurrection-type experiments (i.e. dormant propagules from ancestral populations) are also a promising approach in taxa where genotypes that have experienced varying conditions in the past are available to test responses under experimental conditions^93^. Experimental designs like these outlined above have been criticised for over-simplifying ecological processes, however taking an experimental approach will be essential to tease apart the relative effects of multiple stressors, either simultaneously, or sequentially, or at different stages of an organism’s life-history. Starting with experimental designs or studies at single biological levels is tractable yet will enable refining hypotheses and study designs for the future study of other biological levels in more complex conditions.

Fourth, eco-evolutionary and environmental Big Data, from the molecular to the ecosystem level, provide a broad and expanding scope, particularly when datasets span space and/or time. At the molecular level, Big Data on genes and genomes (NCBI^94^) and databases of their function (Gene Ontology GO^95^, Kyoto Encyclopaedia of Genes and Genomes KEGG^96^) are rapidly increasing. These databases are designed to be taxonomically comparable, or even species-neutral, to enable transfer of functional annotation (molecular function, biological role and cellular location) or gene network information derived from model organisms to inferred orthologues in newly sequenced species. If the current focus on medical science or morphological characters broadens to encompass functions in response to ecological stimuli^97^, then big genomic data will become an even more useful resource for studying the molecular basis of biological resilience. Similarly, finding the most potent data sources for reconstructing time series into the past still requires innovation, but this approach carries considerable promise for analyses of resilience to changes that have already occurred. For example, abiotic data from the last few decades are now openly available (e.g. CORINE^98^, WorldClim^99^, CHELSA^100^) and big data on species occurrences (GBIF^101^), traits (TRY^102^) and abundances through time^103^ are becoming available at an increasing rate. Collecting data of changes in the deeper past requires continued efforts in digitising physical collections (museum specimens^104^) and application and development of new techniques for data extraction and analysis^82^.

At present, most of the global databases (e.g. those mentioned above) at present contain (partially) non-comparable data, and experimental data are rarely combined with observational data despite potential to increase credibility of conclusions^105^. Leveraging big data across biological levels is challenging as it requires intensive upskilling in data integration^106^ and ideally coordinated platforms for e.g. different ecosystems, communities, or management areas of interest (e.g. ‘ePlant’ platform^107^ for data across multiple levels from *Arabidopsis* and crop plants, or ‘Metascape’^108^ for multiple -omics assays to understand molecular mechanisms). However, as the resolution and density of data increases, and new algorithms that make use of large-scale computational resources become available, the possibilities to find and match comparable drivers-to-biotic-units cases will increase. In the meantime, existing data can be analysed by taking advantage of newly developed methods that minimise biases in unrelated or uncertain data (e.g. Bayesian approaches^109^), or when fully comparable data are available, by using mechanistic models that allow moving beyond correlative analyses (e.g. individual-based models^110^). Artificial intelligence could also begin to be utilised to predict the consequences of ongoing and future change. Although ‘black-box’ neural network approaches are popular, symbolic regression (an approach that finds explicit mathematical formulas to explain linear and non-linear relationships) holds much promise for distiling previously hidden natural laws from available data as it derives simpler and more interpretable equations (e.g. in community ecology^111^). However, an outstanding issue is the need to incorporate measures of sampling effort as unbalanced sampling may lead to incorrect interpretations if not accounted for in analyses^112^ – a problem similar to discriminatory biases in social data applications of machine learning.

Box 2 Investigating biological resilience of salmonid fishes Numerous species and populations of salmonid fishes have been the focus of intensive monitoring and sampling programmes extending across many decades because of their socioeconomic significance and important ecosystem roles (including as keystone species). By combining existing research across biological levels (including genes, cells, populations and ecosystems) and evaluating the next steps within a biological resilience research framework, here we provide a worked example of the value of considering multiple biological levels when investigating an ecosystem-level perturbation.

The Barents Sea ecosystem is being perturbed by rapid increases in fishing pressure and climate change. Long-term ecological and environmental data together with life-history phenotype and genetic information from a population of Atlantic salmon (*Salmo salar*) from northernmost Europe is now being used to determine how this organism is responding via adaptation and shaping the overall resilience of the ecosystem (Box 2 Figure). Population genetic analyses^148^ using a 50-year archive of fish scales have supported the hypothesis that reductions in life-history diversity (i.e. apparent low resistance) were actually an adaptive response to the perturbation^149^. The potential drivers of this response have then been investigated by linking these findings with long-term environmental and salmon prey species ecosystem data. It was discovered that as the abundance of capelin, a fat rich prey, declined so too did the abundance of salmon with a large body size and late-maturing life-history strategy^148^. Molecular biological research has also shown that the large-effect gene linked with the late-maturing life-history strategy and body condition in salmon^150^ has important roles in adipocyte production regulation^151^, thus providing connections about biological resilience processes from genes and cells to populations and ecosystems. This example has implications for fisheries management, as prey species abundance was driven primarily by commercial fishing pressure: capelin is a common protein source in domestic animal (including aquaculture salmon) feed. Thus, research across multiple biological levels demonstrated indirect effects of (capelin) fishing on wild salmon life-history diversity.

To move closer to understanding biological resilience, the next steps include determining how selection acts on life-history traits when undergoing an ecosystem-level perturbation (including epigenetic markers on the genome from changes in cellular function, e.g. ref. ^152^), investigating how population-level demographic changes in salmon (including composition according to life-history traits) scale up to influence other ecological interactions within the Barents Sea ecosystem, and measuring the response curves (i.e. resistance and recovery, Fig. 1) of genetic diversity, demographic variables (i.e. effective population size), and community composition, before, during and possibly after the perturbation (i.e. depending on potential management scenarios to lessen disturbance on the ecosystem). This could be achieved by targeted experimental approaches and by using Big Data from both long-term surveys mentioned above and ancient DNA to determine response to past known ecological disturbance events (see ref. ^153^ for an example of herring population dynamics in response to the ‘first example of industrial fishing’ 800 years ago by the Vikings). Demo-genetic individual-based simulations that bring together data from individuals, populations, and communities (e.g. ref. ^154^) could then be a particularly useful method to link data across biological levels and to forecast future scenarios.

**Figure:**
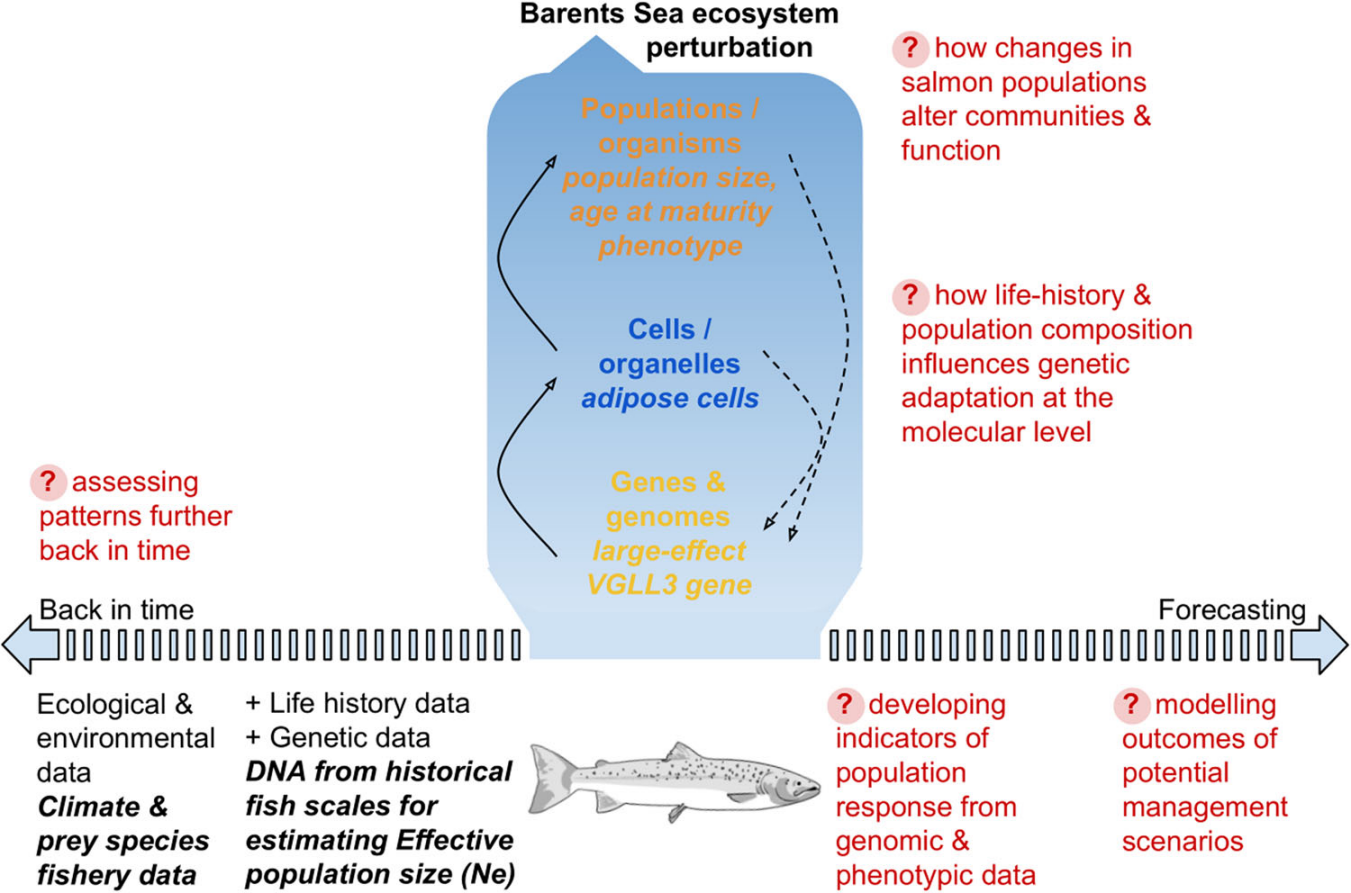
**BOX FIGURE** Overview of an ongoing worked example investigating biological resilience in Atlantic salmon. Text in red indicates next steps for research, see Box text for details.

## Translating biological resilience from research to management and conservation

While there have been many calls to adapt management and conservation of natural resources to improve resilience to environmental change, substantial obstacles remain before this can be realised. First, managers require indicators at levels most appropriate for decision-making. Many of the indicators currently available, however, are system-wide or remain challenging to quantify^15,113,114^. Indicators based on species diversity and habitat connectivity, for example, allow assessment of large-scale patterns^113^, but they are less helpful for management of more tractable system components. Similarly, current discussions around genetic diversity are often difficult to reconcile with ecosystem health as they operate at different timescales and in many cases the links to ecosystem functioning remain unclear (e.g. see ref. ^115^ for a discussion of this problem in the ecological restoration of plants). Second, attempts to manage ‘for resilience’ typically focus on avoiding thresholds or tipping points. Rather, managers need to compare alternative choices, assess potential outcomes with greater certainty than is currently possible, and manage adaptively^8^ (Weise et al., 2020). Third, management approaches largely aim for current or recent known or assumed historical states, rather than attempting to forecast outcomes according to novel future conditions. This is especially problematic when the time horizon is long^8^, for example in forestry and agriculture where long or uncertain time horizons play a large part in the difficulty to translate recommendations^116,117^. Determining how resilience operates at different biological levels has potential to move beyond this stalemate, as the ecological and evolutionary history of components of the system^82,118^ can be used to better evaluate past states, identify more manageable indicators at tractable biological levels, and predict future states under different management scenarios (e.g. Boxs 2, 3).

Box 3 Applying biological resilience Here we highlight the broad potential for the applicability of a biological resilience approach by briefly exploring how it could influence translation and management in two divergent examples: (i) forestry and agriculture, and (ii) human health.

**(i) Biological resilience in forestry and agriculture**

In the past, forest managers have assumed that the climate and other associated factors will remain stable, in spite of the long generation times and individual lifespans of many forest tree species and biomes^117^. However, soil degradation (for example) can occur rapidly compared to the lifespan of the forest and then impact on the ability of trees to withstand other environmental perturbations^155^. Similarly, modern plant breeding selects for yield potential under high and stable resource supply, and generally relies on genetically uniform cultivars. A biological resilience framework, however, encourages a different approach. For example, studies of local adaptation at the population level would help to understand how we can best buffer food and/or timber production against perturbations, perhaps by combining long-term data series and targeted experiments informed by historical farming practices or evolutionary processes^117^. In a context with clear applications for management, Ives and colleagues recently discovered that spatial heterogeneity in crop-harvesting is a major driver of the ecological and evolutionary feedbacks that limit resistance of pea aphids to parasitoid wasps, an important biological control agent^156^. Past perturbations also leave abiotic ‘stress memory’, encoded in DNA methylation and chromatin marks, which may increase resilience over multiple generations^157,158^ in a process of acquired transgenerational resistance^43^. Similarly, interactions across trophic and biological levels are well-known features of plant growth and health, with key work demonstrating that these also influence resilience (e.g. plant-microbe interactions influence resistance to climate change^159^). Harnessing this information could lead to improved crop plant and tree breeding programmes (e.g. ref. ^160^), but much of this work remains embedded in model plant systems, such as *Arabidopsis*. Understanding which features at what biological level are most important to manage (e.g. managing for genetic diversity of monotypic plantations versus diversity of associated mycorrhizal fungi) will require combined approaches and translation of work from model species to natural systems.

**(ii) Biological resilience in human health**

While ecological systems are increasingly becoming viewed as socio-ecological systems^2^, the idea that the human mind and body can be viewed as a complex ecological system is only just beginning to be recognised^123,161^. Understanding how circadian misalignment of sleep/wake cycles leads to a mismatch between abiotic cues and internal cellular functions (e.g. impairment of beta cell function and insulin sensitivity^162^), and then scales up to affect system health via resilience to disease and other stressors, could help to provide more appropriate guidelines for managing shift work, for example. Recent experiences with COVID-19 also demonstrate the need to consider how resilience operates across biological levels: identifying what makes an individual more resilient to a virus at the cellular level (e.g. vaccine development) is not enough if insufficient people take up the vaccine (i.e. population level), or if the virus itself evolves resistance. Indeed, understanding the biological resilience of viral infections, or cancerous growths for example, to medical interventions could assist in progress with treatment. Genetic heterogeneity is known to negatively affect treatment success in cancer^163^, yet this heterogeneity reflects the selective pressures endured, and the variation accumulated, during the whole history of that cancer and can reveal vulnerabilities to therapy^164^. Furthermore, life-history strategies of cells, such as dormancy, can blunt the effects of therapy (e.g. tuberculosis). This suggests that diversity could be an important component of resilience in human health, but this requires testing in translational models.

## Challenges of implementing a biological resilience framework

Here we have argued that understanding and managing for biological resilience requires moving away from the approach of considering function or resilience only at the level of ecosystems, or of focusing studies within a single biological level. We have also stressed how the resilience of the present state not only relies on perturbations experienced in the past (whether contemporary, transgenerational, or deeper in evolutionary time) but that we can also access information about these past responses. Nevertheless, incorporating evolutionary history and complex interactions within and across biological levels is non-trivial, and key challenges exist for modelling complexity and broadening the scope of data collection, as well as setting the temporal and spatial boundaries of the systems or components being studied.

Firstly, in both theoretical and empirical work, we need to identify which connections among what levels are most critical to study. A top-down view of ecosystems works best when considering change over a relatively short period of time, and reduces power for forecasting future responses, either to predicted environmental change or potential management interventions. In ecosystem ecology, species, for example, are normally classified into functional types that leave out valuable information about evolutionary responses to specific perturbations in the past. These responses can however be searched for by mining existing data (e.g. ref. ^33^) or by experiment (e.g. ref. ^30^). Similarly, we need to move beyond research focusing on what makes an individual, or a species, resistant or tolerant to some disturbance event without assessing its relevance to systems or communities. Research in eco-evolutionary dynamics is already beginning to tackle these interactions (see Box 1) and adapting this approach to investigate resilience provides a model for moving forwards. While it is not tractable to measure everything, well-controlled experiments can provide critical data to understand the mechanisms that drive biological resilience – or the lack of it. However, as experiments entail at least some simplification of natural complexity, results will need to be linked conceptually to surveys of the relevant organisms and ecosystems.

Considering multiple levels of biological organisation will also necessitate data collection that tracks responses and maximises phylogenetic, functional, spatial and temporal coverage with minimum monetary cost^119^. This is a challenging task for independent research groups as the acquisition of uninterrupted and consistent time series of ecological and environmental data depend on continued funding. Therefore, coordinated multidisciplinary research projects would enhance data collection and optimise funding streams, making it possible to expand the scope from single- to multiple levels. Some types of data are already available to inform about responses to past conditions, but if we are to make better use of existing and future available datasets, these will require high quality metadata annotations including as many potential ecological variables as possible (and not only the ones directly related to the analyses data were collected for) and easy and open access (e.g. following the FAIR principles^120^).

Providing the evidence necessary to make the case to policy makers is perhaps the most important challenge. For example, accumulating knowledge on ecosystem resilience is yet to change the principles of forestry or cropland management dramatically, which is alarming given that we know many current management practices compromise the ability of future generations to meet their own needs. This may be because resilience is currently difficult to quantify, and a lack of resilience is easier to recognise than a successful management practice. A biological resilience framework could improve identification of ‘resilience indicators’ at scales in which management decisions are made. Tracking genetic diversity at a species level, for example, is a feasible method to collect robust data, and could enable modelling of which actions are likely to be most successful. A critical further step, however, will be improved monitoring of the impact of potential indicators so that we are able to learn from both successful and less successful implementations. Similarly, there are still substantial gaps to bridge between scientists, policymakers and other stakeholders. For example, in commercial farming and forestry widespread adoption of science-led practices depends on short-term economic benefits, so adoption will require policy-based incentives. A deeper understanding of management practices, and co-creation of research questions with stakeholders that will apply management practices, is essential, particularly if we are to implement decisions using an experimental approach.

In summary, biological resilience requires shifting our perspective in eco-evolutionary studies towards investigating terms of resistance versus recovery (the key conceptual outcomes in ecosystem resilience) while also incorporating an eco-evolutionary perspective to better understand ecosystem-level processes (see Fig. 1, Box 1). This requires real multidisciplinary coordinated actions. But we can also begin to take small steps within existing research programmes. Researchers should consider reframing current research to test theory regarding types of responses to disturbance events under study. Or we could consider how influences from evolutionary history may impact ecological responses being detected under current conditions. Although challenging, this approach should provide the advances in data collection, modelling, and testing of hypotheses across levels that are urgently needed to understand and better support resilience in the face of current and future environmental challenges.
